# The Influence of Clamping Pressure on Joint Formation and Mechanical Performance of Ti6Al4V/CF-PEEK Friction-Riveted Joints

**DOI:** 10.3390/ma12050745

**Published:** 2019-03-04

**Authors:** Natascha Z. Borba, Jorge F. dos Santos, Sergio T. Amancio-Filho

**Affiliations:** 1 Helmholtz-Zentrum Geesthacht, Centre for Materials and Coastal Research, Institute of Materials Research, Materials Mechanics, Solid State Joining Processes, 21502 Geesthacht, Germany; natascha.zocoller@hzg.de (N.Z.B.); jorge.dos.santos@hzg.de (J.F.d.S.); 2 Hamburg University of Technology, Institute of Polymer and Composites, 21073 Hamburg, Germany; 3 Graz University of Technology—TU Graz, Institute of Materials Science, Joining and Forming, BMVIT Endowed Professorship for Aviation, Kopernikusgasse 24/1, 8010 Graz, Austria

**Keywords:** Friction Riveting, clamping influence, joint formation, mechanical properties

## Abstract

This work aims at investigating the influence of pre-set clamping pressure on the joint formation and mechanical strength of overlapping direct-friction-riveted joints. A pneumatic fixture device was developed for this work, with clamping pressure varying from 0.2 MPa to 0.6 MPa. A case study on overlapping joints using Ti6Al4V rivets and woven carbon fiber-reinforced polyether-ether-ketone (CF-PEEK) parts were produced. Digital image correlation and microscopy revealed the expected compressive behavior of the clamping system and the continuous pressure release upon the joining process owing to the rivet plastic deformation and the polymer squeezing flow. Two preferential paths of material flow were identified through the alternate replacement of the upper and lower composite parts by a poly-methyl-methacrylate (PMMA) plate—the composite upward and squeezing flow between the parts which induced their separation. The ultimate lap shear forces up to 6580 ± 383 N were achieved for the direct-friction-riveted CF-PEEK overlap joints. The formation of a gap to accommodate squeezed polymer between the composite parts during the process had no influence on the joint mechanical performance. The increase in the clamping pressure for joints produced with a low friction force did not affect the joint-anchoring efficiency and consequently the joint strength. On the other hand, the combined effect of a high-friction force and clamping pressure induced the inverted bell shape of the plastically deformed rivet tip, a lower anchoring efficiency, and the delamination of the composite, all of which decrease the mechanical strength by 31%. Therefore, the higher the friction force and clamping pressure, the more defects would be generated in the composite parts and the more changes in the shape of the plastically deformed rivet tip, leading to a lower level of quasi-static mechanical performance. All the joints failed by initial bearing of the composite and final rivet pull-out. The findings of this work can contribute to further improvement of the clamping design for industrial application.

## 1. Introduction

New concepts and designs of high-performance lightweight structures in a wide range of engineering applications, such as the transportation industry, wind power, and infrastructure, increasingly demand the development of cost-effective, fast, and precise manufacturing and post-processing techniques. The examples include machining [1,2,3], welding [4,5], and joining [6,7,8]. Recently, Saint Jean Industries, a global supplier of parts and subassemblies for automotive and aeronautic industries, has developed a lightweight suspension knuckle made of carbon fiber reinforced polymer (CFRP) and aluminum to increase the part’s stiffness [9]. The joining technology was required to improve and automate the whole manufacturing cycle while guaranteeing the required properties [9]. In such metal–polymer hybrid structures, the post-processing of joining or welding poses challenges due to the high dissimilarities among the properties of the materials. The optimization of such post-processing is, therefore, critical for providing a high quality of components and structures, high productivity, and reproducibility. A typical variable in machining, welding, and joining is the clamping system, which has been reported as a relevant factor to avoid any undesirable distortion and defects and hence a loss of structural integrity [10,11,12,13,14].

The influence of clamping on machining processes is relatively well understood for several materials [15,16,17,18] including fiber-reinforced polymers [10,11,19]. Klotz et al. [11] investigated the influence of the clamping system during the drilling of carbon-fiber-reinforced plastics. The planar specimens were clamped by three and four points as well as by a ring clamping system. The results showed that the distance from the drill axis to the fixed points of the composite significantly influence the process of reactive forces and workpiece quality. By increasing the distance of the clamping points, the workpieces are deflected, leading to typical delamination at the upper composite side, namely “peel-up,” and at the underside, namely “push-out”.

For welding processes of metals, clamping systems have been designed to reduce buckling as well as bending and angular distortions. However, there is still limited literature available on the influence of the clamping on the weld properties. Weidinger et al. [12] investigated the influence of different clamping conditions on laser weld formation and weld strength. The rigid clamping conditions strongly affected the local shrinkage during the weld pool solidification, thereby reducing the solidification cracking and improving the weld quality and strength. According to Richter-Trummer et al. [13], high clamping forces in the order of 2500 N led to better quasi-static mechanical properties of friction stir butt welds of AA2198-T851. Although higher clamping forces induced less distortion and more uniform residual stress distribution through the plate thickness, higher residual stresses were observed in the case of more rigidly clamped parts. The authors also identified a decrease in the degree of separation between the parts during welding by increasing the clamping forces, which may contribute to weld sealing. Shahri et al. [14] reported the influence of clamping on the fatigue life of friction stir welds of AA6005; they showed that the clamping induced local plastic deformation on the crack tip, leading to tensile residual stresses that accelerate the crack initiation during dynamic loading.

Recently, alternative joining technologies suitable for hybrid structures have been developed due to the increasing replacement of conventional metallic materials by polymer composites in aircraft and automotive applications. Such technologies aim to overcome or reduce the drawbacks mainly related to the manufacturing time of traditional techniques such as adhesive bonding and mechanical fastening. Facing this reality, single lap shear joint geometries produced by different innovative joining technologies have been widely explored. However, no previous study has investigated the effect of the clamping system on the joint formation and mechanical performance of composite–composite overlap joints. Goushegir et al. [7] reported the use of a clamping system during and after the friction spot joining (FSpJ) process of AA2024-T3 and carbon-fiber-reinforced polyphenylene sulfide overlap joints. They addressed the importance of clamping to ensure intimate contact between the joining parts and to avoid their separation during the cooling phase as result of different coefficients of thermal expansion of the materials. However, no systematic study has been performed to evaluate the influence of the clamping force on the FSpJ joint properties. A similar study was carried out by Feistauer et al. [6] for ultrasonic joints of Ti6Al4V metal injection molded parts and glass-fiber-reinforced polyetherimide laminates. Although the clamping pressure applied by the sonotrode was shown to influence the joint formation, there is no detailed investigation performed on the correlation between clamping pressure and joint strength. Wagner et al. [20] used a clamping system for the ultrasonic welding of aluminum alloy and CFRP to control the welding force during the joining process, leading to improved joint reproducibility and stable mechanical properties.

Friction Riveting (FricRiveting) is also an alternative joining technology to produce metal-composite overlap joints. The technology relies on the principles of mechanical fastening and friction welding [21]. This technique uses frictional heat and pressure to plasticize and deform a cylindrical metallic rivet into polymer composite parts. In previous publications, the feasibility of the process has been shown on glass- and carbon-fiber-reinforced composites joined with AA6056-T6 [22], commercially pure titanium grade 2 [23], titanium grade 3 [24], and Ti6Al4V [25] rivets. In these works, the main characteristic of the process and the effects of the process parameters on the joint microstructure and mechanical properties were addressed. Amanico-Filho [21] produced friction-riveted overlap joints of the AA2024-T351 rivet and unreinforced polyetherimide (PEI) using a clamping device. Owing to the relatively low strength of AA2024-T351 (tensile strength of 495 MPa [26]), the equilibrium between joining (up to 4.5 kN) and reactive forces during the joining process was not critical, thus resulting in a plastically deformed rivet without any separation of the overlapped PEI parts [21]. Accordingly, no systematic study has been carried out to evaluate the influence of clamping forces on the joint formation and quasi-static mechanical strength. However, by increasing the strength of the rivet base material, higher joining forces (forces up to 20 kN [27]) are needed to achieve sufficient rivet plastic deformation and consequently strong joints. Thus, improved clamping is required to compensate the higher reactive forces and to avoid parts separation [13].

This work aims at investigating the influence of pre-set clamping pressure on the joint formation and mechanical strength of friction-riveted composite overlap joints. A pneumatic fixture device was designed to allow clamping pressure levels up to 1.0 MPa. A case study on overlap joints was produced using an aircraft-applied material combination of Ti6Al4V rivets and woven carbon-fiber-reinforced polyether-ether-ketone (CF-PEEK) plates. The process-related microstructural changes, plastic deformation of the rivet, and the material flow were evaluated using optical and scanning electron microscopy, digital image correlation method, and X-ray computed micro-tomography. The quasi-static mechanical behavior of the friction-riveted joints was evaluated by lap shear testing.

## 2. Principles of Friction Riveting

In Friction Riveting, frictional heat is generated by rivet rotation and insertion into one or more parts, leading to plastic deformation of the metallic rivet tip and its mechanical anchoring. Different joint geometries are possible, including metallic inserting (i.e., rivet insertion into a single polymer part) and overlapping joints (i.e., overlapped parts of unreinforced polymers, reinforced polymers and/or metal). For the overlap geometry, the rivet can be inserted through the upper part with a through-hole and then deformed into the lower part or directly joined within the overlapped parts without holes. For the latter, the process variant is called Direct-Friction Riveting [21,27], which was selected for the present work. Details of the conventional Friction Riveting configuration can be found in [23,28].

**Figure 1:**
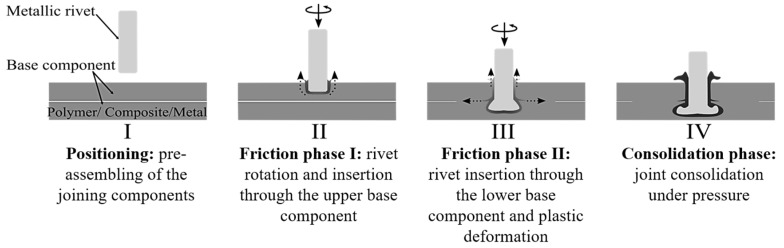
Schematic description of the Direct-FricRiveting process.

Direct-Friction Riveting controlled by force was adopted for this work, with joining phases limited by spindle displacement. Figure 1 depicts the joining phases based on a schematic drawing of the process. After the positioning step (Figure 1I), the rotating rivet reaches a pre-set rotational speed and moves toward the surface of the upper part by applying a constant force (Friction Phase I). Frictional heat is generated, which softens or melts a polymeric layer in the rivet surroundings (Figure 1II). Owing to the continuous insertion of the rivet into the upper part, the softened or molten polymer is expelled as flash outward the joining area (Figure 1II). The second stage of the Friction Phase follows (Figure 1III), in which the rivet is inserted into the lower part at the same rotational speed and higher axial force. The distribution of joint internal stress leads to changes in the material flow, thereby promoting the polymeric squeezing flow (Figure 1III) between the overlapped parts. With constant increase in temperature, the viscosity of the polymer from the lower part decreases concomitantly and the rivet displacement rate increases until the heating phase ends. By this moment, the local temperature at the rivet tip reaches the plasticizing temperature of the metal. The plasticized rivet tip is pressed against the cold polymeric layer underneath it. This provides the required resistance to plastically deform the metal and increases the rivet diameter, thereby anchoring the rivet tip into the lower part. At the end of the Friction Phase II, the rotational speed becomes null, while no further axial force is applied. The joint consolidates by cooling under pressure (Figure 1IV) to avoid the relaxation effects upon cooling, which, in turn, could lead to dimensional changes of the joining parts.

## 3. Materials and Methods

Extruded plane rivets of Ti6Al4V (Henschel KG, Munich, Germany) with a diameter of 5 mm and length of 60 mm were selected. This alloy is widely used for bolts, rivets, and screws in aircraft and devices for the oil and gas industry due to the high specific strength, good corrosion, and creep resistance [29]. Table 1 lists the experimentally determined chemical composition of the titanium alloy. The main properties of this titanium alloy are summarized in Table 2.

The 4.34-mm nominal thickness carbon-fiber-reinforced poly-ether-ether-ketone (CF-PEEK) laminates (Toho Tenax Europe, Wuppertal, Germany) with 58 wt.% nominal fiber content were used as the composite part. The composite laminate consists of 14 plies of carbon-fibers in a stacking sequence of [[(0,90)/(±45)]_3_/(0,90)]_s_. CF-PEEK is a high-performance semi-crystalline thermoplastic composite, which is mainly used in primary and secondary aircraft structures because of its high strength, chemical resistance, and resistance to fatigue failure over aging [30,31]. The main properties of CF-PEEK are summarized in Table 2.

**Figure 2:**
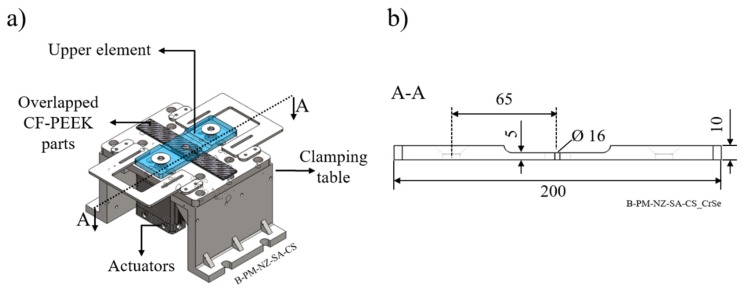
(**a**) Schematic illustration of the pneumatic clamping system and its main elements; (**b**) cross-sectional view of the upper element used to transfer the clamping load. All dimensions are in millimeters.

Friction-riveted single-lap joints were produced using an automated FricRiveting gantry system (RNA, H.Loitz-Robotik, Hamburg, Germany). A pneumatic clamping system built out of low carbon steel with two actuators (DZF-50-25-P-A, FESTO, Islandia, NY, USA), each with a maximum capacity of 1.0 MPa, was used as a sample holder to fix the overlapped composite parts. Figure 2a show the clamping system, while Figure 2b highlights the dimensions of the upper clamping element. A circular fixation diameter of 16 mm was selected to homogeneously distribute the clamping force and reduce the superficial damages of the composite (according to common practices for drilling procedures of fiber reinforced polymers [10]). The selected joining conditions used in this work are listed in Table 3. The combination of joining parameters was obtained from a 2^3^-full-factorial design of experiments with an additional centre point. Owing to the good exploratory power of the regression models acquired for the ultimate lap shear force and the volumetric ratio (R^2^_adj_ = 88% and R^2^_adj_ = 81%, respectively), the assumption of linear dependence between joining parameters and responses was validated. Therefore, in this work, only the maximum and minimum levels of tested joining parameters range were selected, with focus on the effect of internal forces on the joint formation and properties. The individual contribution of the clamping pressure under different joining forces were investigated through two levels of clamping pressure (0.2 and 0.6 MPa) and two levels of friction force (10 and 15 kN), while the rotational speed was constant (15,000 rpm). The results of process optimization and the influence of all process parameters on the joint formation and mechanical strength of friction-riveted joints are not within the scope of this work and will be published elsewhere.

**Figure 3:**
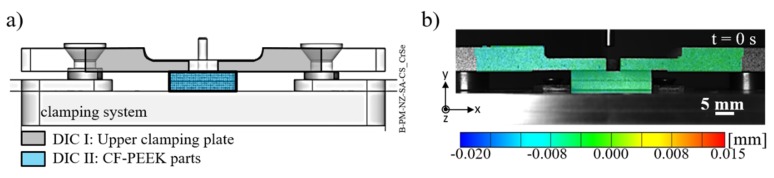
(**a**) Overview of the clamping system showing the areas of analysis for the digital image correlation (DIC) method; (**b**) displacement distribution through the upper clamping plate and the joining parts in the Y-axis direction.

The surface displacement of the clamping device and the specimen during the Friction Riveting process was measured by the digital image correlation (DIC) system (ARAMIS-4m, GOM, Braunschweig, Germany). The displacement and deformation measurements can be used to indicate regions of material under different stress fields—i.e., under compression or tension. The required stochastic speckle pattern on the clamping device and specimen surfaces were prepared using black ink spray paint deposited on a white background. Figure 3a depicts the DIC areas of analysis along with an example of the recorded initial frame (i.e., t = 0 s) which shows the displacement distribution in the Y-axis direction (Figure 3b). An incoherent light source was used to illuminate the DIC areas. A digital camera equipped with 50 mm focal length lens placed perpendicular to the DIC areas was used to record the images. A frame rate of 7 Hz, a facet frame of 15 pixels, and a facet step of 13 pixels, giving an overlap of 2 pixels, were set up in accordance with the image resolution required for accurate results.

Light optical microscopy (LOM; DM IR microscope, Leica, Wetzlar, Germany), scanning electron microscopy (SEM; Quanta^TM^ FEG 650 equipment, FEI, Hillsboro, OR, USA), and X-ray micro-computed tomography, X-ray µ-CT (Y. Cougar- FineFocus X-ray system, YXLON, Hamburg, Germany) were performed to analyze the microstructure of the joints and the fracture surfaces after mechanical testing. The mid-cross-section of the joints was analyzed by LOM to reveal the joint microstructure along with the geometric aspects of the plastic-deformed rivet tip. SEM was used to reveal detailed joint microstructural features and the fracture surface. The samples were prepared following standard materialography procedures. The joints were sectioned through the center of the rivet, embedded in cold resin, ground and polished to obtain a smooth surface finishing. For SEM analysis, the conductivity of the sample surfaces was increased via gold sputtering using a Q150R ES equipment (Quorum Technologies Ltd., Lewes, UK) for 15 s with a current of 65 mA.

**Figure 4:**
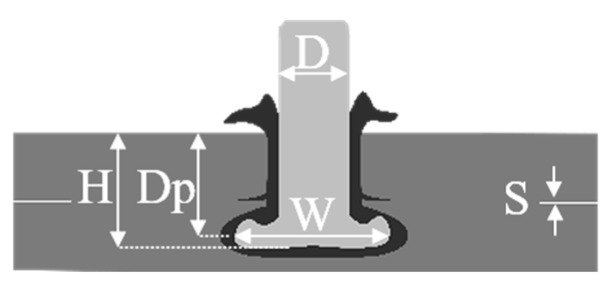
Schematic illustration of a single overlap friction-riveted joint showing geometrical features of the anchoring zone (H, Dp, and W) as well as the separation between the composite parts (S).

From the LOM images, the rivet penetration depth (H), rivet tip width (W), anchoring depth (Dp), and the separation between the composite parts (S) were measured according to Figure 4 and summarized in Table 4. H, W, and Dp were used to calculate the volumetric ratio (VR) (Equation (1)) by adopting the analytical model proposed by Pina et al. [33]. The volumetric ratio uses the interaction volume of the remaining composite material over the deformed rivet tip to represent the anchoring efficiency of the friction-riveted joints.

(1) $$VR=\frac{Dp\times \left(W^{2}-D^{2}\right)}{H\times W^{2}}\;\left[a.u\right]$$

Non-destructive evaluation of friction-riveted joints via X-ray µ-CT was carried out to assess the joint formation over joining time. An operating voltage of 60 kV, a current of 95 µA, and no filters were used for the analyses. The evolution of the geometry of the plastically deformed rivet tip and material flow were investigated by the stop-action procedure. Therefore, joints from four different process steps were produced and evaluated.

**Figure 5:**
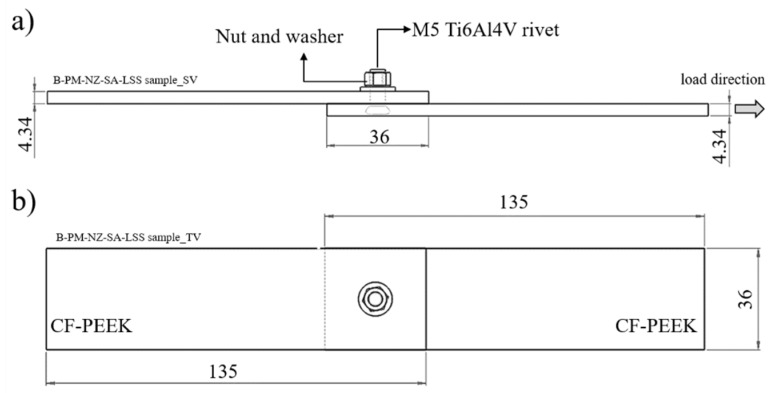
Schematic illustration of single lap shear specimen geometry along with the dimensions and load direction: specimen (**a**) side view and (**b**) top view. All dimensions are in millimeters.

Single-lap shear testing was carried out to analyze the quasi-static joint mechanical performance. The joint strength was evaluated in accordance with ASTM D5961 [34] by using a universal testing machine (model 1478, Zwick Roell, Ulm, Germany) with a load capacity of 100 kN. The transverse test speed was 2 mm/min. Three replicates for each processing condition described in Table 1 were tested at room temperature (21 °C). Figure 5 shows the joint geometry and sample dimensions used in this work. The tightening torque of 5 Nm was applied together with the M5 stainless steel nut and washer to pre-load the joints in order to eliminate any through-thickness failure and to increase the joint load capacity [35]. The stainless steel material for the nut and washer had been selected due to its low static coefficient of friction (µ_e_) when in contact with Ti6Al4V (µ_e_ = 0.36) [36], thereby increasing the pre-load transferability and consequently the tightening efficiency.

## 4. Results and Discussion

### 4.1. Analytical Description of Clamping Force Effect

**Figure 6:**
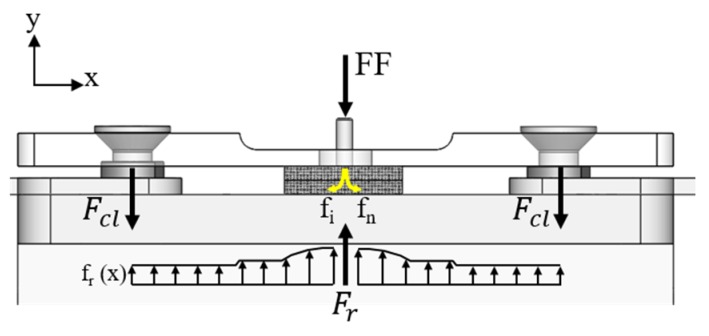
Schematic representation of the forces acting during joint clamping (simplified equilibrium of internal forces).

Figure 6 illustrates the theoretical principle of the clamping concept based on a simplified equilibrium of forces. FF is the friction force, Ʃf_i_ is the sum of internal forces (f_i_) released during the joining process (indicated by the downward arrows), F_cl_ is the clamping force applied by the two actuators, and F_r_ is the resultant force of the distributed reaction over the clamped area (f_r_ (x)). The shear and friction components were neglected for simplifications purposes.

During the joining process, a friction force (FF) is applied to allow the penetration of a rotating metallic rivet through the overlapped composite parts and to contribute to the plastic deformation of the rivet tip. According to Amancio-Filho and dos Santos [37], the distribution of such force over the friction area significantly influences the viscous dissipation in Friction Riveting, which is the main mechanism of heat generation. The friction force is, however, partially lost (∑f_i_) due to the process-related physical changes in the materials, including a decrease in the polymer viscosity and metal plasticizing [24,28]. The reactive load (F_r_) can be expressed using the equilibrium of forces, as analytically presented by Equation (2). Such reaction is responsible mainly for the deformation of the plasticized metal [21] and indirectly for the outward flow of the low viscous polymer from the joining area.

(2) $$F_{r}=FF\;-\overset{n}{\underset{i=1}{\sum }}f_{i}$$

**Figure 7:**
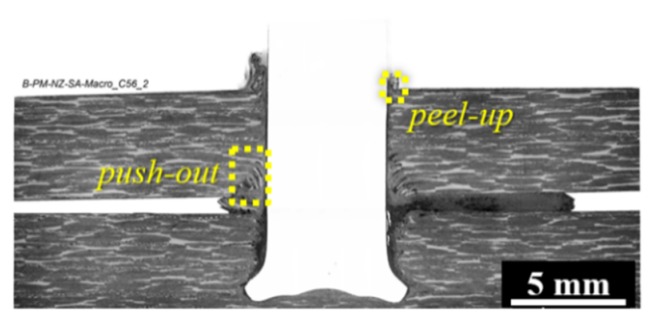
Typical cross-section of a Ti6Al4V/carbon fiber-reinforced polyether-ether-ketone (CF-PEEK) friction-riveted joint produced without the application of external clamping pressure. (Joining parameters: RS: 15,000 rpm, FF: 15 kN, DF: 7.5 mm).

The polymer flow from the lower composite plate and rivet deformation can induce the separation of the composite overlapped parts, which is not restricted by any externally applied forces. Figure 7 shows a typical cross-section of a friction-riveted joint produced without the external clamping device. It can be seen that a significant amount of material, which is squeezed between the composite parts, creates a significant gap between the parts. Moreover, further separation of the composite parts is expected due to the surface delamination evidenced by the fiber peel-up and push-out effects (highlighted by dashed-line squares in Figure 7, a commonly reported phenomenon in the drilling of composites [10,11]. As reported by Matsuzaki et al. [38], this lack of joint sealing can compromise the corrosion behavior and loading capacity of bolted composite joints by creating eccentricities. For butt-friction-stir-welds, the gap between the welded parts leads to differential plate distortion through the welding line, resulting in less ductile welds under tensile and three-point bending loading, as reported by Richter-Trummer et al. [13]. Therefore, any undesirable separation of the joined composite parts should be minimized or avoided.

The effect of F_r_ on the separation of the composite parts is expected to be attenuated by applying external clamping forces (F_cl_) during the Friction Riveting of overlap joints. The resultant force of a distributed clamping load transferred to the joining parts must be equal or superior to the reactive forces from the joining process (Equation (3)) to constrain the upward polymer flow and, consequently, the separation of any plate.

(3) $$2F_{cl}\geq F_{r}$$

Additionally, the clamping scheme may also influence the final shape of the plastically deformed rivet tip and consequently the joint mechanical performance, as will be discussed in Section 4.3. Thus, an optimized balance of the internal and external forces of the Friction Riveting process and the clamping system must be achieved to allow the best compromise between joint formation, quality, and joint mechanical properties. Owing to the complexity of quantifying internal and reaction forces, only the theoretical basis was addressed in this work. Further analysis using the finite element method (FEM) must be carried out to quantitatively prove the established concept.

### 4.2. Evolution of Clamping Displacement and Joint Formation during Direct-FricRiveting

**Figure 8:**
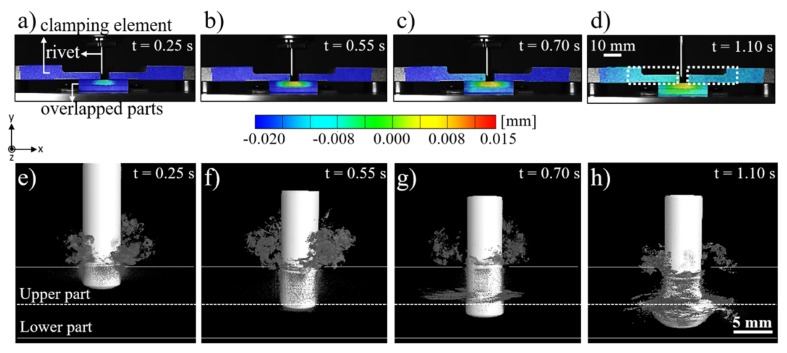
Evolution of the displacement distribution through the clamping system and joint parts (**a**–**d**) along with the joint formation (**e**–**h**) over the process time. (Joining parameters - RS: 15,000 rpm, FF: 15 kN, DF: 7.5 mm, CP: 0.2 MPa).

Figure 8 shows a typical evolution of the clamping and joint displacement at different joining times by the digital image correlation (DIC) method along with the joint formation through X-ray micro-computed tomography. The displacement in the Y-axis provides qualitative evidence of stresses fields through the clamping system and joining parts. In the initial position (t = 0 s, Figure 3b), the clamping system and overlapped composite parts were at 0 mm. As soon as the rivet was inserted into the upper composite part (Figure 8a), the system underwent a compression regime, leading to a negative displacement of the upper clamping element and the composite parts. No initial separation of the composite parts is observed at this stage (Figure 8e). The compression field imposed by the clamping element was maintained over the joining process (Figure 8b,c), decreasing at t = 1.1 s (Figure 8d) when the rivet was released by the chuck, the spindle retracted, and the process finished. This effect is evidenced by brighter colored areas depicted by the dashed-line rectangles in Figure 8d. By this time, the plasticized rivet tip was highly deformed and widened, as shown in Figure 8h. Moreover, throughout the whole joining cycle a localized region in the upper composite plate, in the vicinities of the rivet insertion path, was progressively submitted to a tension field, resulting in a local positive displacement (Figure 8a–d). As previously reported by Altmeyer et al. [24], in the Friction Riveting of CF-PEEK, the process temperatures (415–460 °C) are above the melting temperature of the polymer matrix (T_m_ = 334 °C) and thus melts a thin layer of polymer in the rivet surroundings. This material presents low viscosity and easily flows while the rivet penetrates the composite parts. Therefore, in the initial stages (Figure 8e,f), the molten polymer flowed mainly upwards and thereby formed the so-called flash. Such flow pattern seems to induce the tension field in the rivet surroundings and perhaps an upward bending of the upper composite plate.

**Figure 9:**
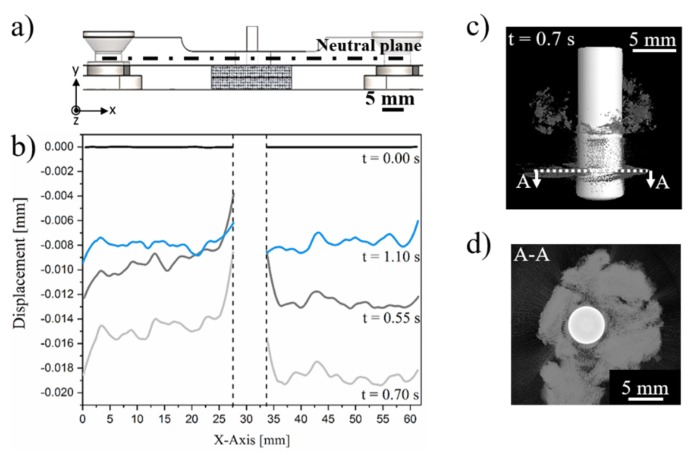
(**a**) Schematics showing the neutral line of the upper clamping element. (**b**) Displacement distribution through the neutral line of the upper clamping element at different joining times. (**c**) X-ray micro-computed tomography showing the side view of the friction-riveted joint at the joining time of t = 0.7 s (A-A is the plane between the composite parts). (**d**) Upper view of the A-A plane that displays the composite squeezing flow between the composite parts (Joining parameters—RS: 15,000 rpm, FF: 15 kN, DF: 7.5 mm, CP: 0.2 MPa).

Figure 9a show the neutral plane of the upper clamping element used to evaluate the displacement evolution over its length and indirectly the compression fields of the DIC areas at different joining times. From Figure 9b, it is clear that partial loss of the clamping compression took place along with the tension created at the surroundings of the hole in the clamping system. Over the joining time, the whole span of the upper clamping element was negatively displaced relative to the Y-axis, reaching a displacement of around −0.02 mm at t = 0.7 s. At this process stage, the distribution of displacement through the length of the clamping element was non-symmetrical to the center. This can be explained by the discontinuous and irregular composite squeezing flow between the composite parts, as presented in Figure 9d. Upon the maximum rivet tip plastic deformation (at t = 1.1 s), the clamping system recovered approximately 50% of the negative displacement and reached values of about −0.009 mm.

**Figure 10:**
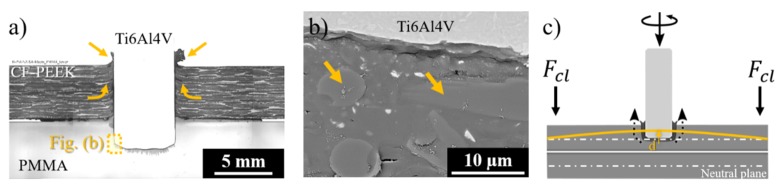
(**a**) Cross-section of the friction-riveted joint with upper CF-PEEK and lower poly-methyl-methacrylate (PMMA) parts. (**b**) Detail of the metal-PMMA interface showing broken fiber embedded in PMMA in the rivet surroundings (**c**) Schematics of the initial joining stage showing possible bending of the upper composite plate and the upward material flow. (Joining parameters—RS: 15,000 rpm, FF: 15 kN, DF: 7.5 mm, CP: 0.2 MPa).

To assess the influence of the stress fields presented in Figure 9 and Figure 10 on the flow of the molten polymer layer, the lower composite part was substituted by a transparent poly-methyl-methacrylate (PMMA) plate. This approach allowed a better observation of the expelled flash material and squeezing flow evolution. Figure 10a shows an overview of the joining area in which the upward flow of the composite is predominant, as indicated by arrows. The SEM image of the PMMA-metal interface (indicated by the dotted-line square in Figure 10a) is depicted in Figure 10b. Figure 9c schematically displays a possible bending of the upper composite plate due to the upward composite flow, promoting localized tension field in the rivet insertion direction, as presented in Figure 9b.

Although the molten composite flowed mainly upward in the initial joining stage, broken fibers embedded into the lower PMMA part were detected underneath and around the rivet tip (arrows in Figure 10b). This clearly indicates that a small amount of the damaged composite from the upper part was transported into the lower part during the initial rivet insertion. Bearing in mind the phenomenological similarities between Friction Riveting and Friction Stir Welding (FSW), the frictional regime of sticking between the welding tool and the plasticized metal being stirred [39,40] may explain the observation. Upon the initial stages of rivet insertion in the upper composite part, the shear stresses at the rivet tip-composite interface may induce a flow of low viscous molten polymer at the same rotational speed as the rivet. This effect leads to composite sticking on the rotating rivet surface, similarly to the metal sticking on the FSW tool [40], driving the upper composite into the lower part. As reported by Schneider et al. [41], such flow can be a complex rigid body rotation, assuming a vortex-ring or a uniform translation pattern. Further investigation using X-ray micro-computed tomography must be carried out to understand such flow patterns for friction-riveted joints. The shear stresses along with the axial joining forces may also lead to the breakage of the superficial composite plies at the overlapped area, thus pushing the solid-damaged composite from the upper to the lower part.

**Figure 11:**
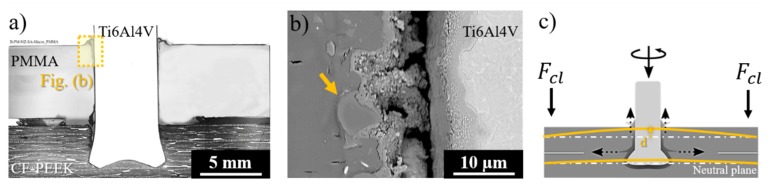
(**a**) Cross-section of friction-riveted with upper PMMA and lower CF-PEEK parts. (**b**) Detail of the metal-PMMA interface showing broken fibers embedded in PMMA in the rivet surroundings. (**c**) Schematics of the final joining stage showing a possible bending of the lower composite plate and the squeezing flow (Joining parameters—RS: 15,000 rpm, FF: 15 kN, DF: 7.5 mm, CP: 0.2 MPa).

The composite flow during the rivet insertion through the lower composite plate is shown in Figure 11. Figure 11a illustrates the cross-section of a joint in which the upper composite part was replaced by PMMA. As it can be observed in Figure 11a and detailed in Figure 11b, broken fibers were detected all over the rivet shaft in the PMMA upper part. Owing to the external restriction imposed by the clamping forces, the molten material from the lower composite plate was mainly driven upward. This flow pattern was absent in the joints produced without any external clamping, whereby the molten material is mainly squeezed out between the overlapped parts (see Figure 7). Although the clamping device restricts the squeezing flow, the rivet plunge and plastic deformation of the rivet tip imposed a positive displacement on the clamping element, as shown at the end of the joining process (Figure 8d). Such a tension field may cause slight bending of the composite parts (see Figure 11c) leading to a material squeeze between the composite parts, the formation of a reconsolidated composite layer, and consequent plate separation. One may assume that the separation of the parts is proportional to the thickness of the squeezed layer. Therefore, it is reasonable to expect that the higher the external clamping force, the more constrained the squeezing flow and the thinner the squeezed layer.

### 4.3. Influence of Clamping Pressure on the Plastically Deformed Rivet Tip and Joint Quasi-Static Mechanical Performance

#### 4.3.1. Process-Related Changes in the Rivet Tip Shape and Joint Microstructural Features

**Figure 12:**
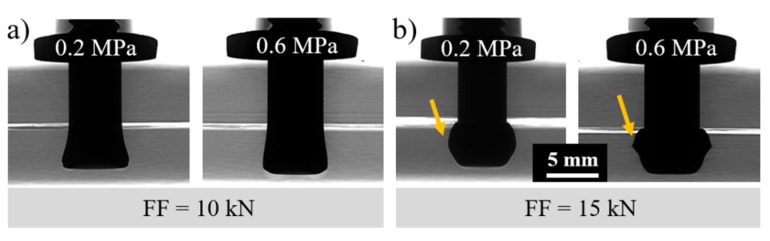
X-ray micro-computed tomography analysis of the joints produced with (**a**) low (10 kN) and (**b**) high (15 kN) friction forces, as well as the influence of clamping pressure on the rivet tip shape. (Joining condition—RS: 15,000 rpm, DF: 7.5 mm).

Figure 12 shows the effect of the clamping pressure on the plastic deformation regime of the rivet tip by X-ray micro-computed tomography. The joints produced with low (Figure 12a) and high (Figure 12b) friction force and constant rotational speed (15,000 rpm) were selected for this purpose. As discussed in Section 4.1, the clamping efficiency depends mainly on the compromise between the internal reactive forces arising from the friction force and the clamping force. Thus, for a lower friction force (FF = 10 kN), less deformation of the rivet tip was evidenced and the effect of clamping pressure did not substantially affect the anchoring zone (i.e., penetration, widening, and shape of the plastically deformed rivet tip). However, when joining with a higher friction force (FF = 15 kN), the increase in clamping pressure from 0.2 MPa to 0.6 MPa induced a higher level of plastic deformation and changes in the rivet tip shape by which the rivet-anchoring zone assumed an inverted bell shape.

**Figure 13:**
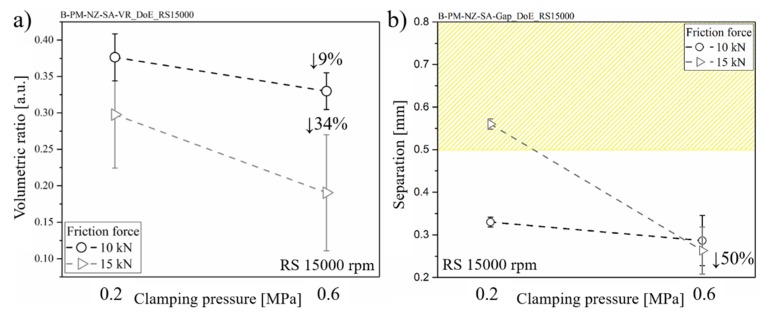
Effect of the clamping pressure on the (**a**) volumetric ratio and (**b**) separation of the composite parts. The hatched upper area in (b) represents the usual range of the sealant and adhesive thickness used for hybrid bolted-bonded joints in aircraft structures [42,43,44].

The rivet-anchoring efficiency was calculated based on the volumetric ratio (Equation (1), Section 3). These results are shown in Figure 13a. By increasing clamping pressure, the volumetric ratio decreases by approximately 34% for higher friction force joints and 9% for lower friction force joints. The combined effect of a higher level of clamping pressure and friction force decreased the composite interaction volume above the deformed rivet tip, thereby decreasing the macro-mechanical interlocking. Therefore, a reduction in the load-carrying capacity can be expected.

Figure 13b shows the influence of clamping pressure on the thickness of the squeezing flow and therefore on the separation of the upper and lower parts. The separation values from 0.20 mm to 0.57 mm were achieved. Such levels are inferior to the common range of adhesive thicknesses (0.5 to 0.8 mm) used in hybrid bonded-bolted composite lap joints for aircraft application [42,43,44]. The squeezing flow between the composite parts in Friction Riveting is assumed to behave similarly to adhesives in hybrid-joining processes, thus contributing to the global joint mechanical performance. As reported by Kelly [44], the hybrid-joining process with flexible adhesives can produce a higher level of joint strength and extend the fatigue life due to improved load transfer distribution through the joint parts.

A 50% decrease in the CF-PEEK plate’s separation was observed for a friction force of 15 kN when the clamping pressure increased from 0.2 MPa to 0.6 MPa (Figure 13b). Under higher clamping pressure, the movement of the joint parts due to high plastic deformation and squeezing flow is constrained and consequently the joining parts are less separated. Such effect is more pronounced for the joints produced under higher friction force (15 kN) towards higher plastic deformation at the rivet tip (see Figure 12) and more composite flow. For a friction force of 10 kN, no significant change in the separation of parts’ was observed by increasing the clamping pressure. In this case, one can assume that the clamping pressure of 0.2 MPa is sufficient to constrain the reduced material flow, inhibiting the movement of the joint parts.

**Figure 14:**
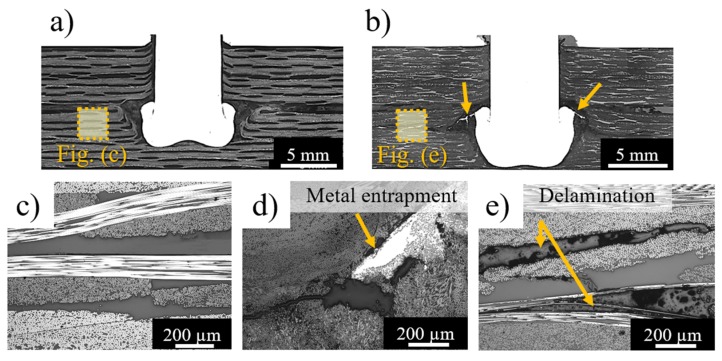
Typical cross-section of joints produced with (**a**) low (CP = 0.2 MPa) and (**b**) high (CP = 0.6 MPa) clamping pressures. (**c**) Detail of the lower composite part where delamination was absent for low CP joints. (**d**) Plasticized metal entrapped between fiber bundles. (**e**) Detail of process-related delamination on the lower composite part for high CP joints. (Joining parameters—RS: 15,000 rpm, DF: 7.5 mm, FF: 15 kN).

Figure 14 shows the process-related delamination in the lower composite part of joints produced with higher friction force (FF = 15 kN) and clamping pressures of 0.2 MPa (Figure 14a,c) and 0.6 MPa (Figure 14b,d,e). The delamination propagates from the joining area when the clamping pressure is increased. As previously explained, the equilibrium between internal reactive and external forces restricts the flow and plastic deformation of the joint materials, imposing preferential paths to the displacement of materials. By increasing clamping pressure, less room for plastic deformation of the rivet tip in the formed hole and between the joining parts is expected. Therefore, the plasticized metal deformed between the bundles of carbon-fiber (highlighted by arrows in Figure 14b and detailed in Figure 14d). Such metal entrapment induces fiber-to-matrix debonding and delamination throughout the lower composite part (Figure 14e). According to Nixon-Pearson and Hallett [45], composite delamination plays a critical role in the quasi-static and cyclic mechanical behavior of conventional bolted composite joints by interacting with bolt clamp-up forces and decreasing the joint structural integrity. Therefore, such process-related defects should be avoided by optimizing the external clamping pressure applied during Friction Riveting, which, nonetheless, is required to minimize the separation of the joining parts and to promote joint sealing.

#### 4.3.2. Joint Quasi-Static Mechanical Performance

**Figure 15:**
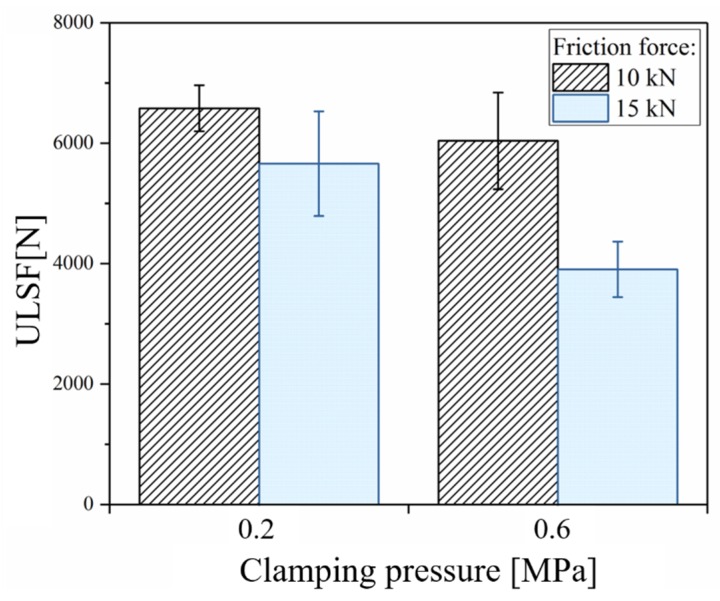
Effect of the friction force and clamping pressure on the ultimate lap-shear force (ULSF) of friction-riveted joints. (Joining parameters - RS: 15,000 rpm, DF: 7.5 mm).

The combined effect of clamping pressure and friction force (FF) on the friction-riveted joint strength is shown in Figure 15. At low FF (FF = 10 kN), increasing the clamping pressure from 0.2 MPa to 0.6 MPa does not display a significant change in the ultimate lap shear force (ULSF) (6580 ± 383 N to 6038 ± 802 N), while a 31% decrease in the ULSF (5660 ± 860 N to 3903 ± 462 N) took place for high FF (FF = 15 kN) joints. As discussed in Section 4.3.1, the increase of clamping pressure (CP) for low FF joints did not affect the volume and geometry of the plastically deformed rivet tip and therefore anchoring efficiency, providing similar joint strength. On the other hand, for high FF joints, changes of CP led to an inverted bell-shape rivet tip and thus a lower level of anchoring efficiency. The shape of the deformation and the low level of anchoring efficiency, along with delamination in the composite part, resulted in weaker joints. Although the separation between joined parts is influenced by CP (see Figure 13), the process-related defects in the composite and the shape and volume of the plastically deformed rivet tip are more compelling for the quasi-static mechanical performance.

**Figure 16:**
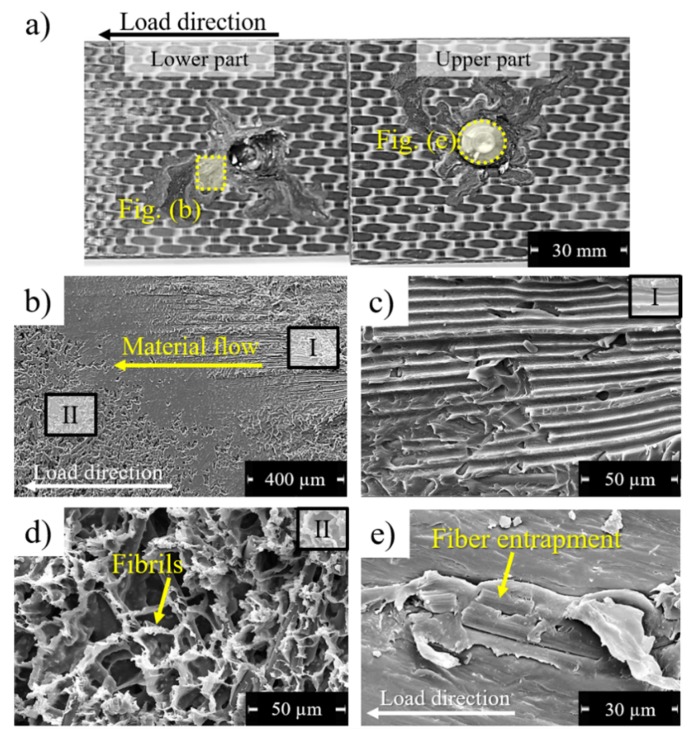
Typical fracture surface of friction-riveted joint. (**a**) General overview of the overlapped area from the lower and upper composite parts. (**b**) Squeezed composite at the surface of the lower composite part, showing the adhesive failure of the reconsolidated composite layer; (**c**) Impressions of fiber tearing in the reconsolidated composite layer; (**d**) Polymer fibrils, indicating ductile fracture of the reconsolidated composite layer. (**e**) Surface of the rivet tip, showing broken fiber entrapment. (Joining parameters—RS: 15,000 rpm, DF: 7.5 mm, FF: 10 kN, CP: 0.2 MPa).

Figure 16a illustrates a typical fracture surface of direct friction-riveted joints. All joints failed by initial composite bearing followed by partial rivet pull-out. Adhesive failure over the reconsolidated composite layer was also identified and is shown in Figure 16b. In such interlayer, fiber tearing impressions (Figure 16c) can be an indication of out-of-plane forces due to secondary bending. Additionally, featureless regions and elongated fibrils (Figure 16d) indicate complex failure micro-mechanisms combining brittle and ductile fractures. Furthermore, Figure 16e depicts the attachment of broken fiber and reconsolidated polymer on the rivet tip surface. Such feature is reported in the literature for friction spot joints (FSpJ) [7,46] as micro-mechanical interlocking, which contributes to the joint mechanical performance. Therefore, one may expect this phenomenon to improve the strength of friction-riveted joints.

## 5. Conclusions

The main goal of the current investigation was to assess the influence of external clamping pressure during the Friction Riveting process on the joint formation and the strength of Ti6Al4V/CF-PEEK single lap joints. The balance of internal and external forces induced by the joining process and clamping system was shown to be relevant to a compromise between joint quality (i.e., minimum defects such as delamination and separation of parts) and high joint strength. The compression imposed by the clamping system varied upon the joining time being partially released at the end of the process when the rivet tip widened and the molten polymer flowed outward the joining area. This compression was also not homogeneously distributed over the span of the clamping element owing to discontinuous and irregular squeezed material between the composite parts. Two preferential paths of the material flow were identified: the composite upward flow, which forms the flash, and squeezing flow between the composite parts, leading to their separation. As expected, by increasing clamping pressure from 0.2 MPa to 0.6 MPa, the squeezing flow was restricted and decreasing the separation of the composite parts was decreased by 50%. Ultimate lap shear forces ranging from 3903 ± 462 N to 6580 ± 383 N were achieved. Despite a 50% decrease in the separation of CF-PEEK parts due to the increasing clamping pressure, no correlation with the quasi-static mechanical performance was observed. A higher level of friction force along with a threefold increase of clamping pressure induced the delamination of the composite part and the inverted bell shape of the plastically deformed rivet tip. These effects led to a 34% loss of the joint-anchoring efficiency and a 31% decrease in joint strength compared to the joints produced under low friction force. All joints failed by initial composite bearing and final rivet pull-out. Considering the similarities between Friction Riveting and the post-processing of composite laminates, the findings of this work may be adopted to further improve and develop the quality and strength of metal-composite overlap hybrid structures through clamping device optimization.

## Figures and Tables

**Table 1:** Chemical composition of Ti6Al4V alloy rivets.

**Weight (wt.%)**	**N**	**H**	**O**	**Fe**	**Al**	**V**	**Ti**
	0.002	0.003	0.107	0.217	6.2	4.5	Bal.

**Table 2:** Properties of the investigated materials.

	Ti6Al4V [29]	CF-PEEK [32]
Tensile/ Shear strength [MPa]	940–1180/550	963 (warp, 0°)/186
E-Modules [GPa]	114	60 (warp, 0°)
Thermal transition [°C]	1655 (Tm)	143 (Tg)/343 (Tm)
Thermal conductivity [W/m K]	17.5	2.0
CLTE [µm/m °C]	9.7 (20–650)	30 (≤ Tg)

**Table 3:** Selected joining conditions of Direct-Friction Riveting.

Rotational Speed, RS [rpm]	Friction Force, FF [kN]	Displacement at Friction, DF [mm]	Clamping Pressure, CP [MPa]
15,000	10.0, 15.0	7.5	0.2, 0.6

**Table 4:** Geometrical features (penetration depth, H; rivet tip width, W; anchoring depth, Dp) of friction-riveted joint measured to calculate the volumetric ratio and the separation (S) between the composite plates.

Joining Condition	H [mm]	W [mm]	Dp [mm]	S [mm]
FF – 10 kN CP – 0.2 MPa	7.6 ± 0.2	6.9 ± 0.1	5.9 ± 0.4	0.3 ± 0.1
FF – 10 kN CP – 0.6 MPa	6.9 ± 0.01	7.2 ± 0.05	4.4 ± 0.3	0.3 ± 0.05
FF – 15 kN CP – 0.2 MPa	7.3 ± 0.2	6.2 ± 0.3	6.2 ± 0.1	0.6 ± 0.1
FF – 15 kN CP – 0.6 MPa	7.5 ± 0.2	5.7 ± 0.2	6.1 ± 0.6	0.6 ± 0.06
